# Self-medication with over the counter drugs, prevalence of risky practice and its associated factors in pharmacy outlets of Asmara, Eritrea

**DOI:** 10.1186/s12889-019-6470-5

**Published:** 2019-02-06

**Authors:** Sirak Tesfamariam, Indermeet Singh Anand, Ghide Kaleab, Samson Berhane, Biruck Woldai, Eyasu Habte, Mulugeta Russom

**Affiliations:** 1 School of Pharmacy, Asmara College of Health Sciences, Asmara, Eritrea; 2 Pharmacology and Clinical Pharmacy Unit, School of Pharmacy, Asmara College of Health Sciences, Asmara, Eritrea; 3 Biostatistics and Epidemiology Unit, School of Public Health, Asmara College of Health Sciences, Asmara, Eritrea; 4 Eritrean Pharmacovigilance Centre, National Medicine and Food Administration, Ministry of Health, Asmara, Eritrea

**Keywords:** Self-medication, Over the counter drugs, Risky practice, Pharmacy outlets, Asmara

## Abstract

**Background:**

Although over the counter (OTC) drugs are believed to be relatively safe, their inappropriate use could have serious implications. The aim of the study was to assess the practice of self-medication, prevalence of risky practice and its associated factors in pharmacy outlets of Asmara, Eritrea.

**Methods:**

A descriptive cross-sectional study was conducted among 609 customers in 20 pharmacy outlets in Asmara between August and September, 2017. Two-stage cluster sampling was employed and data were collected using a structured questionnaire through face to face exit interviews. Descriptive statistics and multivariate logistic regression were performed using SPSS (version 22).

**Results:**

Of the 609 customers, 93.7% had practiced self-medication with OTC drugs; of which 81.8% were at risky practice. On average, each participant was using OTC drugs at least once a month (Median = 1, IQR = 3.67). Educational level (*p* < 0.0001), religion (*p* = 0.047), occupation (*p* = 0.027) and knowledge regarding OTC drugs (*p* = 0.019) were significantly associated with risky practice. Respondents with elementary and below educational level were fifteen times (AOR = 15.49, CI: 1.97, 121.80) at higher risk compared to those with higher education, and students were almost three times (AOR = 2.96, CI: 1.13, 7.73) at higher risk than governmental employees. Furthermore, respondents with below average score in knowledge were more likely to be engaged in risky practice (AOR = 1.83, CI: 1.11, 3.04) compared to those with above average score. The most frequently preferred OTC drug group was analgesics (34.3%) followed by antipyretics (15.7%) and cough and cold preparations (14.2%). About 14% of the respondents admitted that they had taken more than the recommended dose and 6.9% had experienced drug related problems following the consumption of OTC drugs. Always, 35% of the respondents read package insert(s) and 73.9% check expiry dates while purchasing OTC drugs. Refrigerating OTC drugs, where it is not recommended, was also one of the prominent risky practices.

**Conclusions:**

This study revealed that inappropriate self-medication practice with OTC drugs was prevalent requiring early intervention to minimize the risks.

**Electronic supplementary material:**

The online version of this article (10.1186/s12889-019-6470-5) contains supplementary material, which is available to authorized users.

## Background

OTC drugs, that can be purchased by consumers without a medical prescription, are believed to be relatively safe and are appropriate for use without the supervision of healthcare professionals [1]. They are classified according to the WHO Anatomical Therapeutic Chemical (ATC) classification in ten categories as follows: analgesics, laxatives, antithrombotic agents, antacids, cough and cold preparations, antihistamines, dermatologicals, throat preparations, nasal preparations and antidiarrheals [2].

At the community level, good self-medication practices can provide benefits such as saving scarce medical resources from being wasted on minor conditions, controlling chronic diseases, and reducing absenteeism from work due to minor ailments [3, 4]. However, inappropriate self-medication with OTC drugs can have serious implications (including deaths), especially in extremes of ages (pediatrics and geriatrics), pregnant and lactating mothers, and patients with co-morbidities [5–7]. Globally, increasing inappropriate self-medication is seen as a public health concern [5, 8–11].

According to Ranjith et al. as cited by Manohar et al., unregulated or unrestricted availability of OTC drugs is one of the main reasons leading to misuse of these drugs [11]. Moreover, their inappropriate use in developing countries is high due to inadequate knowledge [12, 13], lack of exposure to medical information, inadequate infrastructure [10, 14], and weak laws and regulations [14]. Despite the fact that OTC drugs are used inappropriately and causing drug related problems [8, 15], their number in the market [16] and incidence of their usage are increasing [8, 13].

The list of OTC drugs available in Eritrea (a country in the Horn of Africa), constructed in line with WHO-ATC classification, consists of 55 drugs comprising 68 active ingredients [Unpublished]. These drugs are only available from pharmacy outlets and their presence in shops, groceries, streets and any place other than pharmacy outlet is strictly controlled. Despite the relatively small number of OTC drugs available, common observation of self-medication [12] along with several reported cases is a major concern. For instance, 12.5% of the adverse drug reactions reported to the Eritrean Pharmacovigilance Center were due to OTC drugs, half of which were related to paracetamol and ibuprofen [15].

The aim of the study was, therefore, to assess the practice of self-medication with OTC drugs, prevalence of risky practice and its associated factors. The results obtained are expected to contribute to decreasing the inappropriate use of OTC drugs through changes in the policies and regulations.

## Methods

### Study design and setting

A descriptive cross-sectional study was conducted in 20 selected pharmacy outlets in Asmara (a capital city of Eritrea) between 28th August and 21st September, 2017 for a period of 20 days.

### Source and study population

Residents of Asmara were the source population for this study. As per the Administration of Central Region of Eritrea in 2017, Asmara has a population of 427,183. Customers aged 18 years and above, who visited the pharmacy outlets to purchase medicine(s) during the study and willing to participate were considered as a study population. Customers who couldn’t communicate in either Tigrigna (Eritrea’s national language) or English, who were deaf or mentally unable to communicate were excluded.

### Sampling design

Since the study was done in a community with a large and widely scattered source population, two-stage cluster sampling was used as a sampling design.

First, the list of pharmacy outlets in Asmara was obtained from the National Medicines and Food Administration. Managers of the respective pharmacy outlets were asked to provide estimates of the average number of customers per day. Of the non-systematically arranged 48 pharmacy outlets, 20 sites were randomly selected by probability proportionate to size. The number of subjects to be interviewed at each study site was allocated proportionally to the estimated average number of customers per day. Finally, every 36th customer was interviewed at each study site.

### Sample size estimation

The sample size was calculated by using the single proportion formula without correction for continuity: **n = Z**^2^*P(1-P)/d^**2**^ [17]. The total sample size (n) was calculated using the following assumptions; proportion (P) was taken as 0.5 for no other similar studies were conducted previously, Z statistic for 95% level of confidence (Z = 1.96), degree of precision (in proportion of one, d = 0.05), and 5% non-response rate. Then the sample was adjusted by considering design effect (1.5), as two-stage cluster sampling was used as a sampling design, yielding a final sample size of 609 participants.

### Data collection tool and approach

A structured questionnaire (Additional file 1) consisting of four sections was constructed upon a review of similar studies [8, 10, 18]. The questionnaire was designed to capture data on socio-demographic and background characteristics, knowledge, attitude, and practice of self-medication with OTC drugs.

Knowledge was measured using 10 questions designed to assess the general awareness of the respondents about OTC drugs and their knowledge regarding indications, contraindications, side effects and usage of those drugs. To find association with risky practice, Knowledge was categorized in to two groups (Knowledge below and above average). The median score, which was found to be 15 points, was used to categorize these groups. The maximum possible cumulative score was 20, where questions with one possible answer were scored with one point, and each choice of multiple answer question types was scored individually with one point.

Attitude was measured through 11 questions related to safety, efficacy, accessibility, and usage of OTC drugs in Likert scale. A raw score of attitude was used to find further association, where 55 point was the possible maximum score. Increase in raw score indicates increase in positive attitude.

Finally, Practice was assessed through 20 questions that evaluated the subjects’ health seeking behavior, and safety of their practice. It also included their responses to the reasons that motivated them to self-medicate, drug related problems encountered, and their preference, frequency, and prevalence of OTC drugs used with respect to the ailments intended to be treated.

After explaining the aims of the study and obtaining written informed consent, face to face exit-interviews were done using a structured questionnaire.

### Validity and reliability

Face and content validity of the questionnaire was determined through a panel discussion of experts in the fields of pharmacy, epidemiology, pharmacoepidemiology, public health, and environmental health.

The interviewers were fifth-year pharmacy students trained to ensure intelligibility of the items so as to maximize the within and between inter-rater consistencies.

### Pre-test

A pre-test was conducted among 30 participants on the 9th and 10th of August, 2017, for checking the comprehensibility of the questions at 6 randomly selected pharmacy outlets which were not included in the main study.

After the pre-test, necessary modifications were made in the final version of the questionnaire.

### Statistical analysis

Data were double entered in CSPro (version 7.0) and analyzed using SPSS (version 22). Descriptive analysis of the socio-demographic variables and practice of self-medication with OTC drugs was done using percentages, and median (IQR). Univariate logistic regression was performed to identify the factors associated with risky practice of OTC drugs. Variables found to be significant at the univariate level were considered for multivariate logistic regression to control the effect of potential confounders. Crude and adjusted odds ratios (95% CI) were calculated and all results were deemed to be statistically significant when *p* < 0.05.

### Operational definitions

#### Knowledge above average

Score equal to or above the median of knowledge cumulative score.

#### Knowledge below average

Score below the median of knowledge cumulative score.

#### Risky practice

A respondent was considered to show risky practice if he/she failed to check expiry dates or read labels, took more than the recommended dose, stored OTC drugs improperly, or continued consuming OTC drugs even though he/she noticed unusual color, odor or shape changes.

#### Self-medication

Use of OTC drugs by the consumer to treat self-recognized disorders or symptoms, or the intermittent or continued use of these drugs prescribed by a physician for chronic or recurring diseases or symptoms.

## Results

### Socio-demographic and background characteristics

Out of the 609 participants, 587 completed the interview successfully with a response rate of 96.4%. Three hundred and eighty-one (64.9%) of the respondents were males and 206 (35.1%) were females, out of which 3 (1.5%) were pregnant and 11 (5.3%) were breastfeeding. The median age was 37 years (IQR = 24). Many of the respondents were married (60.1%), aged between 25 and 34 years (26.7%), and governmental employees (37.6%). The educational level was measured in years (attended to formal education) and one-third (33.6%) of the respondents achieved their higher education. Of the respondents, 72.1% had no chronic illness; while Asthma or other COPD were reported in 5.2% of the total cases. The respondents socio-demographic and background information are shown in Tables 1 and 2 below.

**Table 1:** Socio-demographic characteristics of the respondents

Variables	Frequency	Percent
Gender		
Male	381	64.9
Female	206	35.1
Age (Median = 37, IQR =24, Range = 18 to 95)		
18 to 24	113	19.3
25 to 34	157	26.7
35 to 44	118	20.1
45 to 59	118	20.1
60 and above	81	13.8
Marital status		
Married	353	60.1
Single	196	33.4
Divorced	22	3.7
Widowed	16	2.7
Educational level (Median = 12, IQR = 4, Range = 0 to 20)		
Elementary and below	44	7.5
Junior	68	11.6
Secondary school	278	47.4
Higher Education	197	33.6
Occupation		
Governmental services	221	37.6
Private services	83	14.1
Self-employed	134	22.8
Unemployed	29	4.9
Student	47	8
Housewife	73	12.4
Religion		
Christian	504	85.9
Muslim	81	13.8
Others	2	0.3

Religion: Others contain Jehovah’s witness and Atheist

**Table 2:** Background information of the respondents

Variables	Frequency	Percent
Chronic Illness		
No Chronic Disease	429	72.1
Asthma & Other COPD	31	5.2
Diabetes Mellitus	28	4.7
Hypertension	27	4.5
Orthopedic & Rheumatologic problems	14	2.4
Kidney Diseases	14	2.4
Cardiac Diseases	13	2.2
Peptic Ulcer or GI problems	13	2.2
Diabetes Mellitus & Hypertension	10	1.7
CNS disorders	8	1.3
Others	8	1.3
Pregnancy		
Yes	3	1.5
No	203	98.5
Breast feeding		
Yes	11	5.3
No	195	94.7

Chronic Illness: Others contain Allergies, Benign prostatic hyperplasia, Eye problems, HIV, and Psoriasis. *COPD*: Chronic obstructive pulmonary diseases, *GI*: Gastrointestinal, *CNS*: Central nervous system

### Prevalence of self-medication and risky practice with OTC drugs

**Fig. 1:**
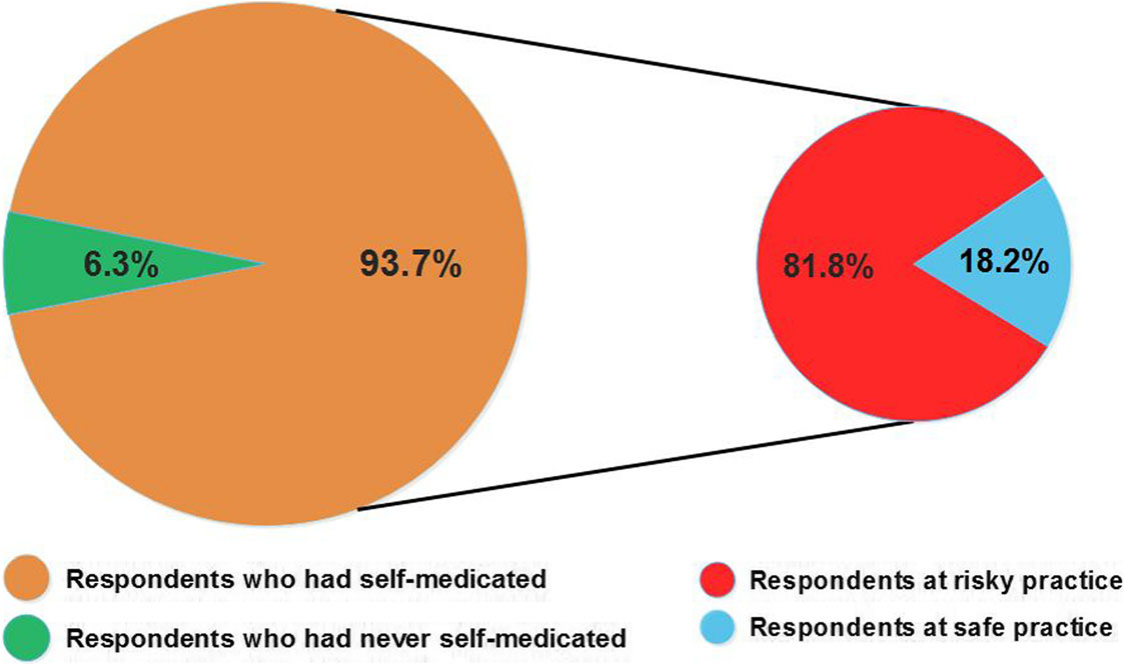
Prevalence of self-medication and risky practice

Almost all respondents (93.7%) stated that they had practiced self-medication with OTC drugs at least once, which includes 65.1% males and 34.9% females. The frequency of self-medication was, on the average, once per month (Median = 1, IQR = 3.67). Out of those who had practiced self-medication, the majority, 450 (81.8%) demonstrated risky practice. This result is summarized in Fig. 1.

### Respondents’ practice of self-medication with OTC drugs

**Fig. 2:**
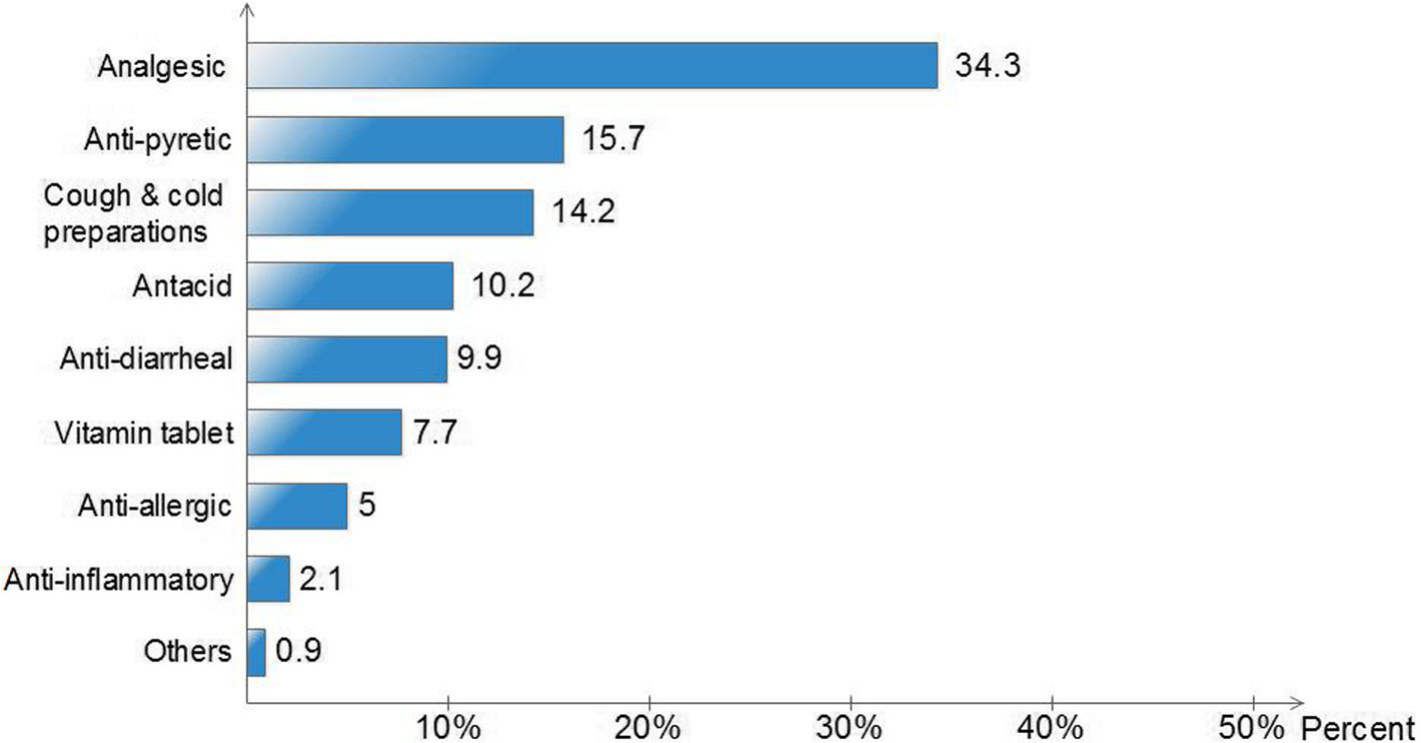
Most preferred drug classes by respondents for self-medication

The majority of respondents reported that they sought information or instruction from pharmacists (34.8%) or medical doctors (27.1%) while self-medicating for the first time. It should be noted that the drugs were not prescribed or suggested by healthcare professionals; rather only information on their use was provided on request. Others (21%) reported that they got advice from friends and relatives, whilst the remainder (3.4%) used the internet or mobile applications as a source of information. The common reasons quoted for self-medicating with OTC drugs were ease of accessibility 290 (34%), saving time 208 (24.4%), perception of being safe and tolerable 125 (14.7%), saving money 48 (5.6%), treating minor ailments 37 (4.3%) and getting quick relief 31 (3.6%). Among OTC drug groups, analgesics, antipyretics, and cough and cold preparations were the most frequently used ones (Fig. 2).

Seventy-nine (14.4%) out of the 550 respondents who had practiced self-medication admitted that they had taken more than the recommended dose at least once. Sixty-five of them took more than the recommended dose to maximize the effectiveness of the medicine, whilst the remaining 14 respondents said that they took more than the recommended dose by mistake.

Of those who had self-medicated, 6.9% had experienced drug related problems following the consumption of OTC drugs. Ibuprofen was perceived to cause the most drug related problems with gastric upset being the major complaint. Almost half of the respondents (44.7%) continued taking the drugs even though they had experienced drug related problems, and around 18% stopped taking the suspected drug(s). But one-third (31.6%) informed pharmacists or other healthcare professionals before doing anything.

**Fig. 3:**
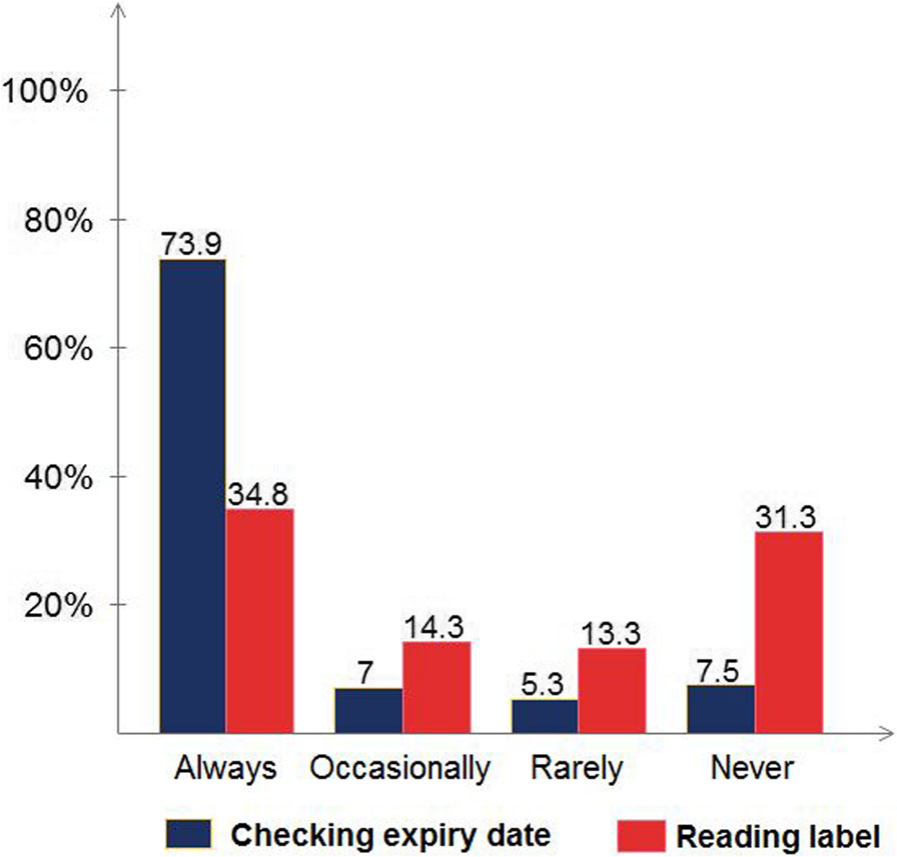
Habit of checking expiry date and reading labels of the respondents

One-third (35%) of the respondents said that they always read package insert(s) and 73.9% said that they always check expiry dates when purchasing OTC drugs (Fig. 3). Around 31%; however, admitted that they never read anything and 7.5% never checked expiry dates.

The majority of respondents (64.9%) reported that they visited health facilities for further diagnosis and treatment if the self-medication failed to work. Other reported decisions include: doubling the normal dose (11.5%), changing to other more powerful OTC drugs (6.9%), taking some time until the symptoms subside (5.3%), repeating the same medication (2.9%) and using home-remedies or recreational activities (8.6%). Similarly, the majority of respondents (70.7%) claimed that they discarded their OTC drugs if they observed changes in shape, color, or odor. Others either consulted pharmacists (8.9%) or would continue to take the drug until it reaches its expiry date (6.2%).

Three hundred and four (46.7%) of the consumers reported that they stored their medicines in dry and cool places where children and direct sunlight could not reach them. Others stored their medicines in a refrigerator (30.9%), on open tables in bedrooms (18.1%), kitchens (1.5%), bathrooms (1.4%) or other places.

### Factors associated with the risky practice

The analysis of factors that possibly affect the risky practice are displayed in Table 3. Educational level, occupation, religion, level of knowledge, attitude, and frequency of self-medication were significantly associated with the risky practice at the univariate level. But, with the adjustment of potential confounders through multivariate logistic regression, educational level (*p* < 0.0001), religion (*p* = 0.047), occupation (*p* = 0.027) and knowledge (*p* = 0.019) were the only variables significantly associated with risky practice. Protective effect of educational level on risky practice was observed and with an increase in the level of education, there was a reduction in the odds of risky practice (Table 3). Respondents who completed junior (AOR = 4.26, CI: 1.66, 10.88) and secondary school (AOR = 4.10, CI: 2.35, 7.15) were four times at higher risk than the reference category, higher education. More distantly, respondents with elementary and below educational level were fifteen times (AOR = 15.49, CI: 1.97, 121.80) at higher risk compared to those in higher education. Muslims were two times (AOR = 2.40, CI: 1.01, 5.72) at higher risk than Christians and students were almost three times at higher risk (AOR = 2.96, CI: 1.13, 7.73) compared to governmental employees. Knowledge regarding OTC drugs was also significantly associated with the risky practice. Respondents with below average score in knowledge were almost two times (AOR = 1.83, CI: 1.11, 3.04) at higher risk than those having a score above average.

**Table 3:** Univariate analysis and multivariate analysis on the association between respondents’ socio-demographic characteristics and risky practice of the respondents

Variable	Univariate analysis		Multivariate analysis	
	COR	95% CI	AOR	95% CI
Gender				
Female	1.57	(0.9, 2.53)	–	–
Male	*Ref*			
Age				
18 to 24	1.8	(0.85, 3.82)	–	–
25 to 34	1.39	(0.70, 2.73)	–	–
35 to 44	1.54	(0.75, 3.17)	–	–
45 to 59	1.32	(0.65, 2.68)	–	–
60 and above	*Ref*			
Marital status				
Single	1.44	(0.89, 2.32)	–	–
Divorced	5.62	(0.74, 42.49)	–	–
Widowed	4.01	(0.52, 30.90)	–	–
Married	*Ref*			
Educational level				
Elementary and below	21.67*	(2.91, 161.24)	15.49**	(1.97, 121.80)
Junior	5.15***	(2.11, 12.58)	4.26**	(1.66, 10.88)
Secondary school	4.51***	(2.75, 7.40)	4.10***	(2.35, 7.15)
Higher education	*Ref*		*Ref*	
Occupation				
Private service	1.75	(0.89, 3.42)	1.18	(0.57, 2.45)
Self-employed	2.1*	(1.15, 3.72)	1.05	(0.54, 2.02)
Unemployed	1.9	(0.62, 5.74)	0.82	(0.25, 2.72)
Student	2.18	(0.87, 5.45)	2.96*	(1.13, 7.73)
Housewife	3.56**	(1.45, 8.70)	1.45	(0.54, 3.88)
Governmental service	*Ref*		*Ref*	
Chronic Illness				
Yes	0.8	(0.5, 1.28)	–	–
No	*Ref*			
Religion				
Muslim	2.42*	(1.08, 5.44)	2.40*	(1.01, 5.72)
Christian	*Ref*		*Ref*	
Knowledge of OTC drugs				
Below average	2.36***	(1.48, 3.78)	1.83*	(1.11, 3.04)
Above average	*Ref*		*Ref*	
Attitude towards OTC drugs	0.9***	(0.85, 0.95)	0.94	(0.88, 1.00)
Frequency of self-medication per month	1.06*	(1.01, 1.11)	1.03	(0.98, 1.08)

Note: *** = *p* < 0.001, ** = *p* < 0.01, * = *p* < 0.05, Ref = *reference category*

## Discussion

Even though in Asmara, OTC drugs are strictly controlled to be sold in pharmacy outlets only, the self-medication with OTC drugs was found to be prevalently accompanied by high risky practice. The prevalence of self-medication is similar to an Indian study [10] and the prevalence of inappropriate practice is consistent with the findings from a study conducted in Bahrain [19] but much higher than reported by a Nepalese study [8]. Because of difference in the target population among the Indian, Bahraini and the current study, caution should be exercised during result comparison.

In the current study, most of the respondents (73.9%) reported that they always check expiry dates before taking OTC drugs. This result emphasizes that the respondents were very cautious about using expired drugs. This result is similar to a previous study conducted in Eritrea [12], but higher than the result of a Nepalese study where only 9.1% always check expiry dates [8]. On the other hand, the reported habit of reading the package insert before using medicines was very low. Some of the major complaints by the respondents were that package inserts are not available for almost all OTC drugs and the available ones are not prepared in the local language which makes it difficult to comprehend. In addition to the individuals’ behavior, the above result might be lowered by the unavailability of package inserts. Other studies showed that in Italy, but conducted among teenagers, 40% did not read the patient information leaflet last time they took OTC drugs [20]. In descending order, in Nepal 48.2% [8], Ethiopia 45.5% [13], India 25% [11] read leaflets carefully. Moreover, overdosing for the intention of better treatment outcomes and storing medicines in inappropriate places (prominently in a refrigerator) were identified as major risk practices in the customers of pharmacy outlets.

Understanding the sources of information is helpful in designing interventional scheme to promote safe self-medication practice. In our study, despite the most referred source of information being pharmacists, engrossingly, the prevalence of risky practice was high. Observed poor communication bridge between customers and pharmacists could contribute in producing such a controversial result. Comparatively, a higher percentage of pharmacists’ involvement in self-medication was observed in studies done in Italy [20], Nepal [21], Ethiopia [13], and Sri Lanka [22]. Easy accessibility of OTC drugs was reported as the main reason that encouraged the respondents to self-medicate. But, in studies done in Palestine [23], India [24], Nepal [25], Egypt [26], respondents self-medicated because they believed that the condition was not serious enough to seek medical attention. Among OTC drug groups, analgesics were the most frequently preferred analogous to studies conducted in Bahrain and India [19, 27].

In the present study, 14.4% of the respondents admitted that they had taken more than the recommended dose which is much lower than studies conducted in Nepal (86.4%) [8] and USA (33%) [28]. Most of the respondents (82%) took more than the recommended dose to maximize the effectiveness of the medicine which is consistent with that of the USA (91%) [28]. The majority of respondents, either continued or stopped taking the suspected drug(s) to cause drug related problem before consulting pharmacist or other health care professionals. This implies that there is a lack of knowledge towards reporting side effects to concerned authorities. It is evident that disseminating information to the community on how to report side effects could increase awareness, thereby indirectly decrease complications of drug related problems. The majority of respondents reported that they visited health facilities for further diagnosis and treatment if self-medication failed to work and discarded OTC drugs if they noticed changes in shape, color, or odor. The above-stated results were satisfactory which needs to be empowered.

Educational level, religion, occupation, and knowledge were identified as risk factors for inappropriate use of OTC drugs. As anticipated, a positive effect of educational level and knowledge on risky practice was observed. In a study done in Nepal [8], a similar significant association between educational status and practice of self-medication with OTC drugs was reported. Additionally, there was no significant association between age and practice of self-medication in both studies. Due to unknown reason, the difference in self-medication practice between Christians and Muslims was significant with a border case *p*-value (*p* = 0.047). Thus, this can be one avenue for further research to uncover the reasons involved, if there are any. Gender, marital status, and chronic illness categories of the socio-demographic characteristics were found not to affect the risky practice significantly at both univariate and multivariate level of association.

### Strengths and limitations

An adequate sample size considered as fairly representative study participants with a high response rate was the major strengths of the study. It is also assumed that the findings of this study can be generalized to our source of population.

Causality assessment was not run for the reported drug related problems owing to the limitation of the study. Thus, the reported drug related problems might not reflect the actual situation in the field. The questionnaire constructed to measure the knowledge and attitude of the respondents was not psychometrically validated. Besides, as patients were asked to provide history of use and adverse effects, recall bias might affect the data accuracy.

## Conclusions

In summation, inappropriate use of OTC drugs in the Asmara community was found to be of concern requiring further research and stricter control on the use of OTC drugs for self-medication. Refrigerating OTC drugs, where it is not recommended, was one of the prominent risky practices that warrant immediate attention. The reported habit of checking expiry dates before taking OTC drugs was satisfactory, but the habit of reading the package insert was very low. As a remedial action to elevate the habit of reading package inserts, the Ministry of Health of Eritrea should legislate laws concerning the preparation of patient information leaflet in a local language for easy comprehensibility. Low educational level, poor knowledge, religion and occupational status of the respondents (students) were significantly associated with the risky practice of self-medication with OTC drugs. Truth to this nature, health education on medication through different media outlets and in health facilities has paramount importance to promote the safe/appropriate use of OTC drugs.

## Additional file

Additional file 1: Questionnaire for obtaining the knowledge, attitude and practice of self-medication with Over the Counter drugs among pharmacy outlet customers in Asmara, Eritrea. (PDF 327 kb)

## Abbreviations

AOR: Adjusted Odds Ratio
ATC: Anatomical Therapeutic Chemical
CI: Confidence Interval
COR: Crude Odds Ratio
CSPro: Census and Survey Processing System
IQR: Interquartile range
OTC: Over the Counter
SPSS: Statistical Package for Social Sciences
WHO: World Health Organization
