# Supplementary material for: Self-medication with over the counter drugs, prevalence of risky practice and its associated factors in pharmacy outlets of Asmara, Eritrea
Source: BMC Public Health. 2019 Feb 6;19:159. doi: 10.1186/s12889-019-6470-5 (PMC6364400; doi:10.1186/s12889-019-6470-5)
Supplement: Supplementary file 1 — Questionnaire for obtaining the knowledge, attitude and practice of self-medication with Over the Counter drugs among pharmacy outlet customers in Asmara, Eritrea. (PDF 327 kb) [file 12889_2019_6470_MOESM1_ESM.pdf]

Questionnaire - English Version

Questionnaire for obtaining the knowledge, attitude and practice of self-medication with Over the Counter drugs among pharmacy outlet customers in Asmara, Eritrea

SECTION A: SOCIO-DEMOGRAPHY

Instruction: Circle, put tick/cross, or write the answer that corresponds with your best answer.

No	Socio-demographic questions		Answer	Skip
**1.**	Sex		1. Male<br>2. Female	
**2.**	Age in years			
**3.**	Marital status		1. Married<br>2. Single<br>3. Divorced<br>4. Widowed	
**4.**	Highest level of education			
**5.**	Occupation		1. Governmental<br>2. Private service<br>3. Self employed<br>4. Unemployed<br>5. Student<br>6. House wife	
**6.**	Chronic Illness			
**7.**	Pregnancy		1. Yes<br>2. No<br>3. Not applicable	
**8.**	Lactation		1. Yes<br>2. No<br>3. Not applicable	
**9.**	Religion		1. Christian<br>2. Muslim<br>3. Other, *please specify*<br>________________	
**10.**	Ethnic group			
**11.**	Nationality			

Section B: Knowledge

Instruction: Circle your answers on the choices provided at the right side.

No	Questions about knowledge	Answer	Skip
**1.**	Medicines are always used on the prescription of a doctor.	1. Yes<br>2. No<br>3. Don't know	
**2.**	All Over the Counter drugs are safe and effective.	1. Yes<br>2. No<br>3. Don't know	
**3.**	Over the Counter drugs are used for treating diseases like:	1. Hereditary diseases<br>2. Minor illnesses and injuries<br>3. Don't know	
**4.**	Over the Counter drugs are approved for self-care.	1. Yes<br>2. No<br>3. Don't know	
**5.**	Which of the following drugs fall under OTC drugs? (multiple answers)	a. Antipyretics<br>b. Anti-cold<br>c. Analgesics<br>d. Anti-microbials	
**6.**	Over the Counter drugs could be used after their expiry date.	1. Yes<br>2. No<br>3. Don't know	
**7.**	Over the Counter drugs can:	1. Sometimes cause side-effect(s)<br>2. Mostly cause side-effect(s)<br>3. Never cause side-effect(s)<br>4. Don't know	
**8.**	While using Over the Counter drugs, caution should be taken mostly in: (multiple answers)	a. Pregnancy<br>b. Lactation<br>c. Elderly<br>d. Children<br>e. Adolescent/middle adults	
**9.**	If suspected side-effect(s) are seen, then one should: (multiple answers)	a. Immediately stop using the drug<br>b. Take low dose until side effect(s) subside<br>c. Continue taking the drug regardless the side effect(s)<br>d. Report to a Doctor or Pharmacist<br>e. Other, *please specify*<br>____________________	
**10.**	All Over the Counter drugs when taken along with prescribed drug are safe.	1. Yes<br>2. No<br>3. Don't know	

SECTION C: ATTITUDE

Instruction: For the following section, the choices are represented by numbers in which **1=Strongly disagree, 2=Disagree, 3=Neither agree or disagree, 4=Agree, and 5=Strongly agree.** Circle or tick √ **ONE** answer that best matches your general opinion.

No	Statements	Strongly Disagree	Disagree	Neither Agree or Disagree	Agree	Strongly Agree
**1.**	Over the Counter drugs that are used for self-medication are safe.	1	2	3	4	5
**2.**	Over the Counter drugs are cheaper and convenient.	1	2	3	4	5
**3.**	Paracetamol in overdose is a powerful poison.	1	2	3	4	5
**4.**	Over the Counter drugs can modify or alter the action of another drug.	1	2	3	4	5
**5.**	All Over the Counter drugs can be used in case of pregnancy.	1	2	3	4	5
**6.**	Pain killers when taken on an empty stomach does not cause gastritis.	1	2	3	4	5
**7.**	Over the Counter drugs are not affected by storage conditions, like temperature, moisture and direct sunlight.	1	2	3	4	5
**8.**	Liquid medicines could be used when opened after one month.	1	2	3	4	5
**9.**	Eye/Ear drops could be used after one month of opening.	1	2	3	4	5
**10.**	It is better I don't take Over the Counter drug when I am ill.	1	2	3	4	5
**11.**	Over the Counter drugs are safe but would seek a physician advice before using it.	1	2	3	4	5

SECTION D: PRACTICE

Instruction: Circle your answers on the choices provided at the right side.

No	Questions regarding Practice	Answer	Skip
**1.**	Have ever practiced self-medication with Over the Counter drugs?	1. Yes<br>2. No →	Skip to Q. 18
**2.**	With whom did you consult before using Over the Counter drugs? (multiple answers)	1. Pharmacist<br>2. Doctor<br>3. Friends/relatives<br>4. Leaflet<br>5. Internet and mobile applications<br>6. Other, *please specify* ____________	
**3.**	When did you consume Over the Counter drugs?	1. When symptoms are minor/manageable<br>2. Whenever I feel sick<br>3. When I can't visit doctor<br>4. Other, *please specify* ____________	
**4.**	Common reason(s) for using Over the Counter drugs is: (multiple answers)	1. Time saving<br>2. Low cost<br>3. Safe and well tolerable<br>4. Easy accessibility<br>5. Other, *please specify* ____________	
**5.**	Which categories of medications are mostly preferred by you for self-medication?	1. Antipyretic<br>2. Cough and cold preparation<br>3. Analgesics<br>4. Anti-inflammatory<br>5. Anti-diarrheal<br>6. Antacids<br>7. Vitamin tablets<br>8. Anti-allergic<br>9. Other, *please specify* ____________	
**6.**	Have you ever taken Over the Counter drug(s) more than the recommended dose?	1. Yes<br>2. No →	Skip to Q. 8
**7.**	Why did you take more than the recommended dose?		
**8.**	Have you ever experienced adverse effect from Over the Counter drugs?	1. Yes<br>2. No →	Skip to Q. 12
**9.**	What was the side-effect(s)?		
**10.**	After what medication was the side-effect(s) experienced?		
**11.**	What did you do after the side-effect(s)?		

**12.**	How often do you read the instructions on drug's label before use?	1. Always<br>2. Occasionally<br>3. Rarely<br>4. Never	
**13.**	How often do you check the expiry date?	1. Always<br>2. Occasionally<br>3. Rarely<br>4. Never	
**14.**	What do you do in case Over the Counter drug don't work well?	1. I double the dose<br>2. I change other powerful Over the Counter drug<br>3. I go to health facility<br>4. Other, *please specify*<br>__________________	
**15.**	If Over the Counter drugs showed change in shape, color, odor:	1. Immediately discard the drugs<br>2. Continue using till it expires<br>3. Continue using even if after it expires<br>4. Other, *please specify*<br>__________________	
**16.**	Where do you store Over the Counter drugs?	1. Medicine box<br>2. Bed room / open in the table<br>3. Refrigerator<br>4. Kitchen<br>5. Bathroom<br>6. Other, *please specify*<br>__________________	
**17.**	What is the average number of Over the Counter drugs that you are using per month?		
**18.**	Type of the medication purchased	1. OTC drug<br>2. Prescription Only Medicine →<br>3. Both	End of Interview
**19.**	Name of the purchased OTC drug		
**20.**	Ailment want to be treated		

Thank you very much for completing this questionnaire!
